# Immunoproteasome impairment via β5i/LMP7‐deletion leads to sustained pancreatic injury from experimental pancreatitis

**DOI:** 10.1111/jcmm.16682

**Published:** 2021-06-15

**Authors:** Laura L. de Freitas Chama, Frédéric Ebstein, Birthe Wiesrecker, Preshit R. Wagh, Elke Hammer, Frank U. Weiss, Heike Junker, Maja Studencka‐Turski, Markus M. Lerch, Elke Krüger, Matthias Sendler

**Affiliations:** ^1^ Department of Medicine A University Medicine Greifswald Greifswald Germany; ^2^ Institute of Medical Biochemistry and Molecular Biology University Medicine Greifswald Greifswald Germany; ^3^ Department of Functional Genomics Interfaculty Institute for Genetics and Functional Genomics University Medicine Greifswald Greifswald Germany; ^4^ German Center for Cardiovascular Research Partner Site Greifswald Greifswald Germany

**Keywords:** cell death, cytokines, ER stress, pancreatitis, PSMB8

## Abstract

Uncovering potential new targets involved in pancreatitis may permit the development of new therapies and improvement of patient's outcome. Acute pancreatitis is a primarily sterile disease characterized by a severe systemic inflammatory response associated with extensive necrosis and a mortality rate of up to 24%. Considering that one of the reported disease mechanisms comprises the endoplasmic reticulum (ER) stress response and that the immunoproteasome is a key regulator to prevent proteotoxic stress in an inflammatory context, we investigated its role in acute pancreatitis. In this study, we demonstrate that immunoproteasome deficiency by deletion of the β5i/LMP7‐subunit leads to persistent pancreatic damage. Interestingly, immunoproteasome‐deficient mice unveil increased activity of pancreatic enzymes in the acute disease phase as well as higher secretion of Interleukin‐6 and transcript expression of the Interleukin IL‐1β, IFN‐β cytokines and the CXCL‐10 chemokine. Cell death was increased in immunoproteasome‐deficient mice, which appears to be due to the increased accumulation of ubiquitin‐protein conjugates and prolonged unfolded protein response. Accordingly, our findings suggest that the immunoproteasome plays a protective role in acute pancreatitis via its role in the clearance of damaged proteins and the balance of ER stress responses in pancreatic acini and in macrophages cytokine production.

## 1 INTRODUCTION

Acute pancreatitis, one of the most common gastrointestinal diseases, is an inflammation of the pancreas, thought to be primarily due to premature intra‐acinar activation of digestive pancreatic zymogens.^1^, ^2^ Although most patients present with a mild form of the disease, about 20% develop severe pancreatitis associated with organ dysfunction, requiring intensive care.^3^ The global incidence of acute pancreatitis has been reported by Xiao et. al.^4^ to be 34 cases per 100 000 per year. Autodigestion of the pancreas by its own proteases leads to acinar cell injury and subsequently to a local and systemic inflammatory response.^5^, ^6^ Nevertheless, paradoxical results from trypsinogen deficient mice, which still developed experimental pancreatitis,^7^, ^8^ have brought this traditional concept under review and suggest that the molecular mechanisms associated with the disease onset and progression are not fully understood. Many studies, specifically in animal models, have identified a variety of cellular events as being involved in the pathogenesis, such as premature activation of pancreatic enzymes,^9^ increase in calcium signalling,^10^ inflammatory cell infiltration,^11^, ^12^ mitochondrial dysfunction^13^ and endoplasmic reticulum stress (ER stress).^14^, ^15^

Acini are the major source of digestive enzymes, thereby exhibiting the highest rate of protein synthesis and folding capacity with more abundant ER then all other cell types.^16^ ER stress is caused by accumulation of misfolded or unfolded proteins, which may arise during synthesis, folding and secretion of secretory and cell‐surface proteins. Therefore, elucidating the molecular mechanisms of protein homeostasis may identify potential new targets of acinar cell injury. ER stress is counterbalanced by the unfolded protein response (UPR) machinery, which has a dedicated role in protein quality control and homeostasis, interacting with degradation pathways such as autophagy and the ubiquitin proteasome system (UPS).^17^ Impairment of UPR was found to be one cause of genetic varieties of pancreatic disorders.^18^, ^19^

The UPS plays an important role in intracellular protein degradation and turnover in eukaryotes via a multi‐enzymatic machinery, entailing target protein ubiquitination and subsequent proteolysis by the 26S proteasome.^20^ The immunoproteasome is an isoform of the constitutive proteasome (26S proteasome), which is assembled upon a triggering inflammatory response by Toll‐like or cytokine receptor signalling, type I or type II interferons (IFNs), and TNF‐α releases. It arises from the replacement of the β‐catalytic subunits β1, β2 and β5 in the 20S standard proteasome core by β1i (large multifunctional peptidase 2, LMP2 encoded by *Psmb9*), β2i (multicatalytic endopeptidase complex‐like‐1, MECL‐1 encoded by *Psmb10*) and β5i (LMP7 encoded by *Psmb8*), respectively, during de novo proteasome formation.^21^, ^22^ Over the years, the immunoproteasome has been recognized as an important player in shaping innate and adaptive immune responses by degradation of inflammatory mediators and improved MHC class I antigen presentation.^23^

In addition to its roles in innate and adaptive immune cells, the immunoproteasome has also been linked to non‐immune functions, namely cell differentiation and protein homeostasis, including intracellular protein clearance^24^ and subsequently, may thus control of the ER stress levels. Likewise, a growing body of evidence has unveiled a consistent interdependence between ER stress imbalance and the immune system.^25^, ^26^ In view of the immunoproteasome role for protein clearance and ER stress pathways, and the association of human mutations with autoinflammatory diseases,^27^ our aim in this work was to investigate the role of the β5i/LMP7 subunit in acute pancreatitis.

## 2 MATERIALS AND METHODS

### 2.1 Animals

Experiments were performed using male and female β5i/LMP7^+/+^ (littermate controls) and β5i/LMP7^−/−^ mice C57BL/6J strain background, generated as previously reported,^28^ weighing 20 to 25g at about 2‐3 months of age. Both genotypes were bred and maintained at the Greifswald University Animal Care Facility. Food and water were provided ad libitum to the animals, which were kept in a controlled environment with a constant 12:12‐hours light‐dark cycle.

### 2.2 Caerulein‐induced acute pancreatitis model

Acute pancreatitis was induced by eight hourly intraperitoneal injections of caerulein (50 µg/kg bodyweight; cat: C9026‐Sigma, Merck), as previously described.^29^ The experimental protocol was approved by the institutional Animal Care and Use Committee. Three experimental groups were designed: control animals (0 hour; no caerulein injections), 8 hours corresponding to the acute phase of the disease and 24 hours to the recovery phase. Five independent experiments were performed, and no difference regarding sex was statistically significant. Serum and tissues were collected, processed and adequately stored to the experimental procedures, as described ahead.

### 2.3 Biochemical assays

Quantification of serum activities of amylase and lipase enzymes were assayed by photometric assays kits from Roche Hitachi. Lactate dehydrogenase (LDH) was measured in serum using a specific kit assay from Sigma (cat: MAK066, Merck). Pancreatic enzymatic activities, such as trypsin and chymotrypsin, were determined in tissue homogenates as previously reported.^30^ Proteasome Chymotrypsin‐like activity assay was also performed in pancreas homogenates, as described in supplementary method. Myeloperoxidase (MPO) activity measurement in lung was performed as previously described.^11^ All activities from tissue homogenates were suitably normalized by protein concentration using Pierce BCA protein assay (cat: 23227, Thermo Fisher Scientific).

### 2.4 Measurement of pro‐inflammatory cytokines

The IL‐6 pro‐inflammatory cytokine was measured in serum and supernatant samples by fluorescence activated cell sorter (FACS) analysis using the CBA mouse inflammation kit, according the manufacturer's instructions (cat: 552364; Becton Dickinson). IL‐1β cytokine was measured in supernatant from macrophage culture using Peprotech kit assay (cat: 900‐M47).

### 2.5 Imaging analyses

Histological pancreatic damage was verified by haematoxylin and eosin (H&E) staining in paraffin‐embedded pancreas (2 µm sections) previously fixed in 4% formaldehyde. To detect apoptosis, we used ApopTag^®^ Red In Situ Detection Kit (cat: S7165‐Sigma; Merck) and acinar cell proliferation by immunohistochemistry for the nuclear localization of Ki67 (rabbit polyclonal, cat: IHC‐00375; Bethyl).^31^ For the immunofluorescence experiments, we prepared frozen sections (2 µm) of Tissue‐Tek^®^ OCT compound‐embedded pancreas. A list of all used antibodies can be found in supplementary methods. Quantification of the CD68 and Ly6G immune staining was estimated manually from 5 random pictures in a magnification of 200‐fold from each animal. Total number of cells, through WEKA Segmentation in Image J software, was used to obtain the percentage of positive cells. The same design was applied to calculate apoptosis ratio.

### 2.6 Isolation of acini

Pancreas were carefully removed from wild‐type C57BL/6J, β5i/LMP7^+/+^ (littermate controls) and β5i/LMP7^−/−^ mice and digested with 1 mg collagenase (Collagenase from Clostridium histolyticum; EC.3.4.24.3; SERVA Electrophoresis), under sterile conditions, as initially described.^32^ Cell suspension was resuspended in fresh medium without collagenase, and after 30 minutes of resting in water bath at 37℃, three different approaches were addressed: 1) protease activation was examined in living cells upon an enzymatic kinetic reaction up to 60 minutes with 1 µM Cholecystokinin (CCK, fragment 26‐33; C‐2175 Sigma) stimulation whereas necrosis was measured by propidium iodide exclusion^33^; 2) acini were directly platted in 12 well plates and incubated with 1 µM CCK for 6 hours or 8 hours and 24 hours in a humidified atmosphere of 5% CO_2_ at 37ºC, for subsequent total RNA or protein extraction; 3) or challenged with the 1 µM of CCK for 30 minutes prior addition to the macrophage culture, as described below.

### 2.7 Co‐culture of acini and macrophages

Femur of wild‐type C57BL/6J, β5i/LMP7^+/+^ (littermate controls) and β5i/LMP7^−/−^ mice were collected under sterile conditions. Bone marrow stem cells were differentiated into macrophages (BMDM), as previously established.^34^ At the day of the experiment, BMDM culture received fresh medium, before being exposed to isolated acini for 6 hours or 9 hours. Supernatant was collected for cytokine measurements, and the cells were washed with PBS to withdraw non‐adherent cells. Subsequently, cells were harvested with 5 mM EDTA in PBS on ice and resulting pellet stored at −80ºC for posterior total RNA extraction.

### 2.8 Transcript expression by quantitative Real‐time PCR

All reagents were purchased from Thermo Fisher Scientific, unless otherwise specified. Pancreas, acini and macrophages were collected and total RNA was extracted with Trizol reagent (cat: 15596026). RT‐qPCR analyses are described in supplementary methods; detailed primer sequences are shown in Table 1. GAPDH or 5S ribosomal RNA expression was used as internal control.

**TABLE 1:** Murine primer sequences for the transcripts assessed by quantitative real‐time PCR

Transcript	Sequences [5'‐ 3']	
	Forward	reverse
BiP	TGGATAAGAGAGAGGGAGAGAG	CACCACTTCAAAGACACCATTG
ATF4	TCGAATGGATGACCTGGAAAC	AATTGGGTTCACTGTCTGAGG
sXBP‐1	TGGAAGAAGAGAACCACAAACT	CATTCCCAAGCGTGTTCTTAAC
CXCL‐10	GTGTTGAGATCATTGCCACG	AAGGAGCCCTTTTAGACCTT
IFN‐β	ATCCAAGAGATGCTCCAGAATG	CCAGGAGACGTACAACAATAGTC
IL‐1β	GAGGACATGAGCACCTTCTTT	GCCTGTAGTGCAGTTGTCTAA
IL‐6	CCAGAGTCCTTCAGAGAGATACA	CCTTCTGTGACTCCAGCTTATC
LMP7	ATCGAGATTAACCCTTACCTGC	AGATGCGTTCCCCATTCC
5S	GCCCGATCTCGTCTGATCTC	GCCTACAGCACCCGGTAT TC
GAPDH	CCACTCACGGCAAATTCAAC	CTCCACGACATACTCAGCAC

### 2.9 Measurement of protein levels by Western blotting

Protein extracts from pancreas homogenates and acini normalized for protein concentration (10 µg protein) were subjected to SDS–PAGE and then transferred onto PVDF (Polyvinylidene fluoride) or nitrocellulose membranes (Thermo Fisher Scientific) for immunoblotting; a detailed protocol is described in supplementary methods. Band intensities were quantified by densitometry using the Image J software (https://imagej.nih.gov/ij/) and normalized by loading control (amylase in case of acini and Ponceau staining for pancreas extracts).

### 2.10 LC‐MS/MS measurements and data analysis

A total of 7 μg of protein resuspended in 8 M urea/ 2 M thiourea was prepared and digested with trypsin using a bead‐based SP3 protocol.^35^ Details on mass spectrometric and data analysis are compiled in Supplementary methods. Differential abundance was calculated as the median of all possible pairwise peptide ratios calculated between replicates of all connected peptides. Proteins with an adjusted *P*‐value <.05 were considered as differentially abundant. The mass spectrometry proteomics data have been deposited to the ProteomeXchange Consortium via the PRIDE [1] partner repository with the dataset identifier PXD024514.

### 2.11 Statistical analyses

Data are presented as means  ± SEM, and the number of independent experiments is indicated. Statistical analyses, significant when *P* < .05, were performed with GraphPad software (GraphPad) using one‐way analyses of variance (ANOVA) followed by Turkey's multiple comparison test or unpaired Student's *t* test, as appropriate. For the Supplementary Figure 1, SigmaPlot 11.0 (Systat Software) was used for the analyses.

## 3 RESULTS

### 3.1 The β5i/LMP7 subunit is up‐regulated in acute pancreatitis

**FIGURE 1:**
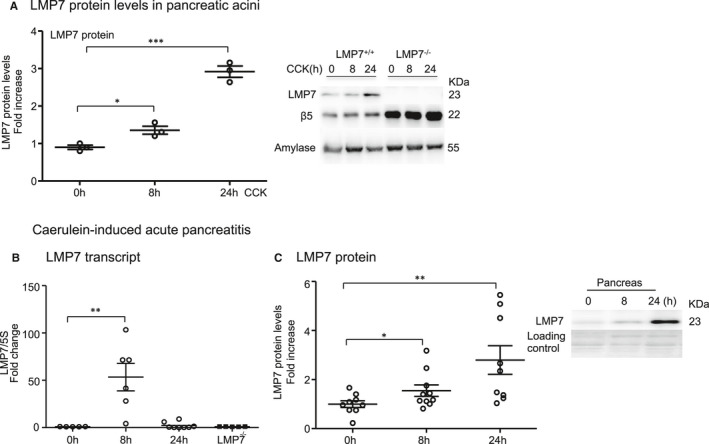
Expression and regulation of LMP7 subunit in isolated acini and in experimental acute pancreatitis. A, Isolated acini were treated with 1 µM of CCK for 8 h and 24 h. The levels of LMP7 and β5 subunits were determined by Western blotting. Amylase was used as an internal control, and a ratio of LMP7 was calculated. B, Transcript levels of LMP7 were evaluated by real‐time PCR in the pancreas after caerulein‐induced pancreatitis. 5S was used as an internal control (n = 5‐8). C, Western blotting analyses of LMP7 subunit in the pancreas during pancreatitis (n = 9‐10). Data from independent experiments are expressed as means ± SEM. *P < .05, **P < .01, ***P < .001

First, we determined whether β5i/LMP7 subunit and its constitutive counterpart β5 were expressed and differentially modulated in an in vitro model of pancreatitis using isolated acini. After supramaximal stimulation of the cells for 8 hours and 24 hours with CCK, we assessed the protein levels of both subunits by Western blotting. Pancreatic acini express basal levels of both subunits but only the β5i/LMP7 subunit was up‐regulated after 24 hours of treatment (Figure 1A). Of note, immunoproteasome‐deficient acini up‐regulate the standard β5 subunit most likely to compensate for decreased chymotrypsin‐like activity (Figure 1A). In a next step, we investigated whether the regulation of the β5i/LMP7 subunit would also be shifted in an in vivo mouse model of pancreatitis. We therefore used the caerulein model, which induces a mild and reversible form of the disease.^29^ Three experimental groups were designed: control (0 hour), acute phase (8 hours) and recovery phase (24 hours). Interestingly, the β5i/LMP7 transcript levels were significantly increased at 8 hours after the onset of pancreatitis. At 24 hours, its levels returned to baseline as measured in the control group (Figure 1B). Importantly, no β5i/LMP7 transcripts were detected in the pancreas from β5i/LMP7^−/−^ mice. Western blot analysis of pancreas homogenates showed an approximately threefold increase in β5i/LMP7 protein levels at 24 hours compared to basal levels (Figure 1C).

### 3.2 Higher pancreatic damage in β5i/LMP7‐deficient mice

**FIGURE 2:**
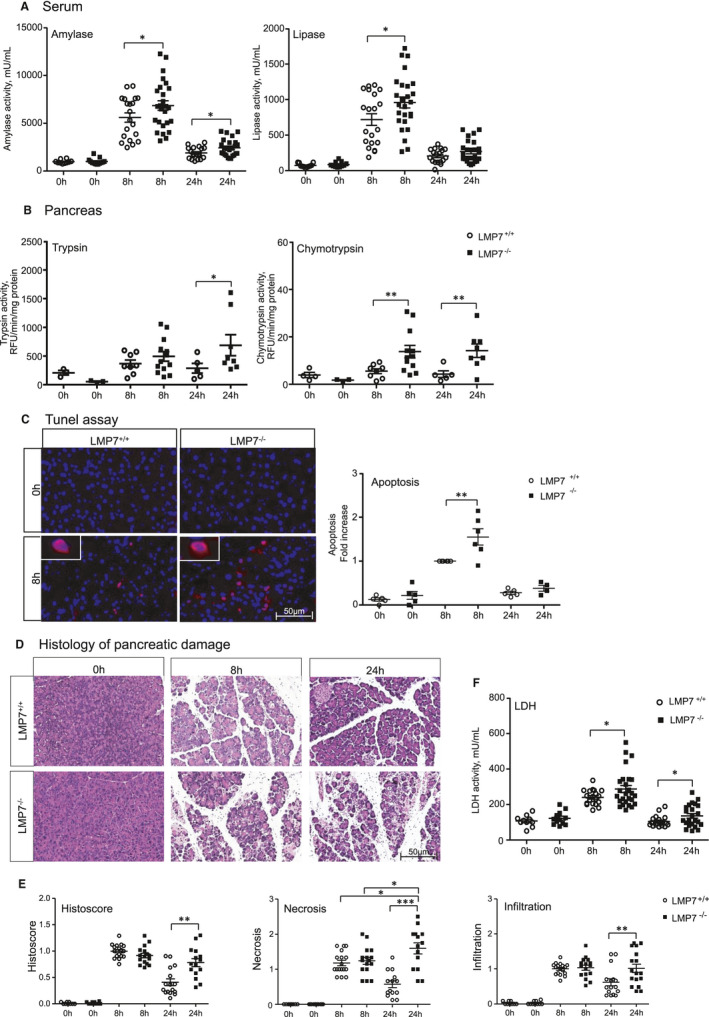
Higher pancreatic damage in the absence of LMP7. A, Serum amylase and lipase were measured using specific chromogenic substrates. 0 h (n = 10‐13), 8 h and 24 h (n = 20‐25). B, Trypsin and chymotrypsin activities were quantified in the pancreas using corresponding fluorescent substrates. 0 h (n = 3‐4), 8 h (n = 8‐13) and 24 h (n = 5‐9). C, Apoptotic bodies were detected by tunel DNA fragmentation assay in paraffin‐embedded pancreas, and DAPI was used as nuclei staining (n = 4‐6). Apoptosis ratio was computed based on positive versus total number of cells, normalized by the corresponding LMP7^+/+^ 8 h group. D, Histology of pancreatic damage was visualized by haematoxylin and eosin staining of pancreas. E, Histoscore of disease severity, including necrosis and infiltration. Image representative of five independent experiments. Scale bars, 50 µm. Size of experimental groups: 0 h (n = 9‐10), 8 h and 24 h (n = 14‐17). F, Serum lactate dehydrogenase activity detected in all experimental groups. 0 h (n = 10‐13), 8 h and 24 h (n = 20‐25). Data obtained from five independent experiments are expressed as means ± SEM. *P < .05, **P < .01, ***P < .001

To investigate the role of the β5i/LMP7 immunoproteasome subunit in pancreatitis, suggested by its up‐regulation in vivo, disease severity was evaluated in β5i/LMP7^−/−^ and littermate controls β5i/LMP7^+/+^ mice. We performed the caerulein‐induced pancreatitis model, including the same experimental groups as before. Biochemical markers of pancreatic injury,^11^ such as serum amylase and lipase activities, were increased by about 20% in β5i/LMP7^−/−^ mice (Figure 2A). Additionally, we measured trypsin and chymotrypsin activities in the pancreas homogenates of littermate controls and β5i/LMP7^−/−^ mice. Trypsin and chymotrypsin consistently showed higher activities in the absence of β5i/LMP7 (Figure 2B). To examine early protease activation of living acini towards stimulation in the absence of β5i/LMP7, we performed an ex‐vivo enzymatic assay.^30^ In the time‐course of 1 hours CCK treatment, activities of trypsin and cathepsin B were similar in both genotypes, as well as the necrotic rate (Supplementary Figure 1). Thus, we showed that the lack of β5i/LMP7 did not alter the early protease activation within acini but during later pancreatitis progression.

Since intracellular protease activation is associated with acinar cell death,^30^ we next investigated whether apoptosis and necrosis were likewise increased in β5i/LMP7^−/−^ mice. Apoptosis quantification was accomplished by labelling DNA fragmentation (Tunel assay) in paraffin‐embedded pancreas. We observed that β5i/LMP7^−/−^ mice exhibited a significantly higher number of Tunel‐positive staining, approximately 1.5‐fold higher at 8 hours, compared to littermate controls (Figure 2C). Histological pancreatic damage was further verified by haematoxylin and eosin staining,^11^ allowing us to identify and score necrotic areas and leukocytes infiltration. Pancreas from β5i/LMP7^+/+^ and β5i/LMP7^−/−^ mice exhibited equivalent pancreatic damage in the acute phase of the disease (8 hours). However, necrosis was increased at 24 hours in β5i/LMP7‐deficient mice and the tissue damage persisted for a longer period compared to the littermate group (Figure 2D,E). Pancreatic injury was approximately twofold higher in the absence of β5i/LMP7 at the 24 hours time point (Figure 2E). To assess cell death, we measured lactate dehydrogenase (LDH) activity in serum, which was also increased by 20% in β5i/LMP7^−/−^ mice at 8 hours (Figure 2F). Of note, increased inflammatory cell infiltration was detected in the absence of β5i/LMP7 (Figure 2E), leading to the next question to be addressed.

### 3.3 β5i/LMP7 deficiency correlates with increased inflammation

**FIGURE 3:**
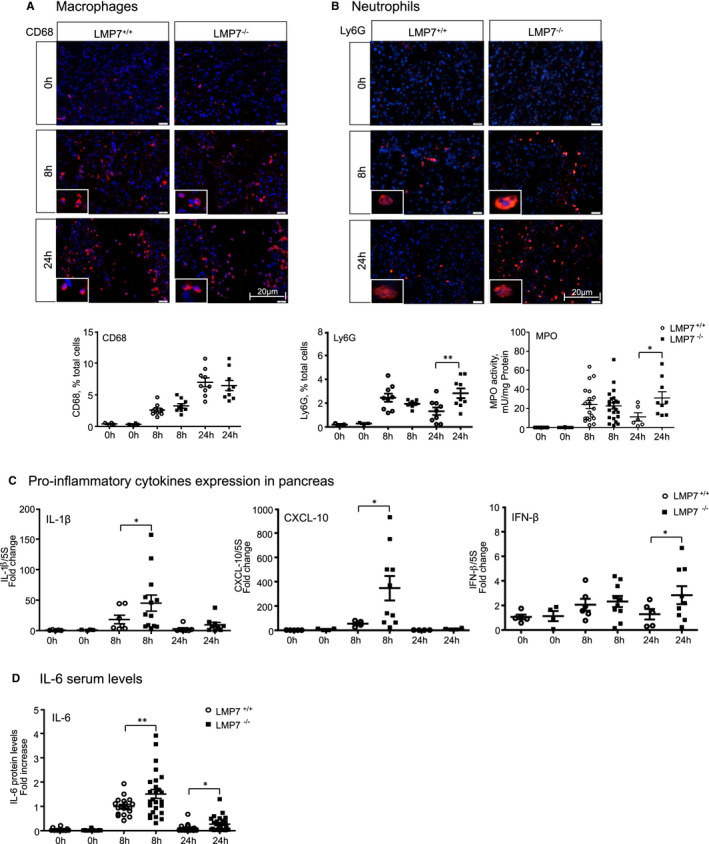
Increased inflammation in the absence of LMP7. Immunofluorescence in frozen pancreas sections was performed using macrophages (CD68; A) and neutrophil (LY6G; B) markers antibodies. Quantification of positive cells for each leukocyte population was calculated based on the total number of cells, visualized by DAPI nuclei staining (CD206 data not shown). 0 h (n = 3‐4), 8 h and 24 h (n = 8‐9). B, MPO activity detected in lung using chromogenic substrate. 0 h (n = 12‐13), 8 h (n = 19‐23) and 24 h (n = 6‐9). C, Transcripts levels of IL‐1β, CXCL‐10 and IFN‐β genes were determined by real‐time PCR using specific primers with 5S as housekeeping. LMP7^+/+^ groups—0 h (n = 4‐5), 8 h and 24 h (n = 4‐7); LMP7^−/−^ groups—0 h (n = 4), 8 h (n = 10‐13) and 24 h (n = 4‐9). D, Interleukin‐6 levels were measured in serum by CBA assay. Data were normalized to the values of the 8 h LMP7^+/+^ group. 0 h (n = 10‐13), 8 h and 24 h (n = 20‐25). Scale bars, 20 µm. Data are representative of five independent experiments and expressed as means ± SEM. *P < .05, **P < .01

Our group has previously shown that acinar cell damage is linked to a pro‐inflammatory state characterized by increased infiltration of macrophages and neutrophils into the pancreas, which correlates with the extension of necrotic areas and decrease of healthy exocrine tissue.^34^ We therefore next characterized these leukocytes population by immunofluorescence in pancreatic tissue from all experimental groups. This approach was performed using specific antibodies for profiling of pro‐ and anti‐inflammatory macrophages phenotypes (CD68 and CD206, respectively) and neutrophils (Ly6g). No significant difference was noticed in the count of infiltrating macrophages (Figure 3A). However, a significant increase in neutrophil population was observed at 24 hours (Figure 3B). Next, we measured the myeloperoxidase (MPO) activity in lungs, which is a peroxidase enzyme abundantly expressed in neutrophil granulocytes.^36^ As expected from the neutrophil infiltration in the absence of β5i/LMP7, we also detected enhanced MPO activity (Figure 3B). We then quantified the activation of pro‐inflammatory mediators in serum and in pancreas. Along with extended acinar cell injury, we found that the transcription of pro‐inflammatory cytokines IL‐1β, CXCL‐10 at 8 hours and IFN‐β at 24 hours was increased in the absence of β5i/LMP7 compared to littermate controls by approximately 2.5‐fold to sixfold (Figure 3C). Likewise, serum levels of circulating IL‐6 cytokine were elevated after 8 hours of pancreatitis (Figure 3D). To analyse whether the alterations in the pro‐inflammatory cytokines regulation originated from pancreatic acini or macrophage BMDM cells, acinar cells were either stimulated with CCK alone or co‐incubated with BMDM cells. This second approach allows the evaluation of macrophages activation in the onset of pancreatitis.^34^ In both cell types, we observed a higher regulation of IL‐6 and IFN‐β cytokines in the absence of β5i/LMP7. The CXCL‐10 chemokine was up‐regulated in pancreatic acini whereas IL‐1β was significantly elevated in BMDM. As seen in isolated acini, β5i/LMP7 subunit was also induced in co‐exposed acini macrophages (Supplementary Figure 2). Taken together, our cytokine data indicate an increase in pro‐inflammatory cytokines in acute pancreatitis in the absence of β5i/LMP7 subunit.

### 3.4 Impairment of ubiquitinated protein degradation in the absence of β5i/LMP7

**FIGURE 4:**
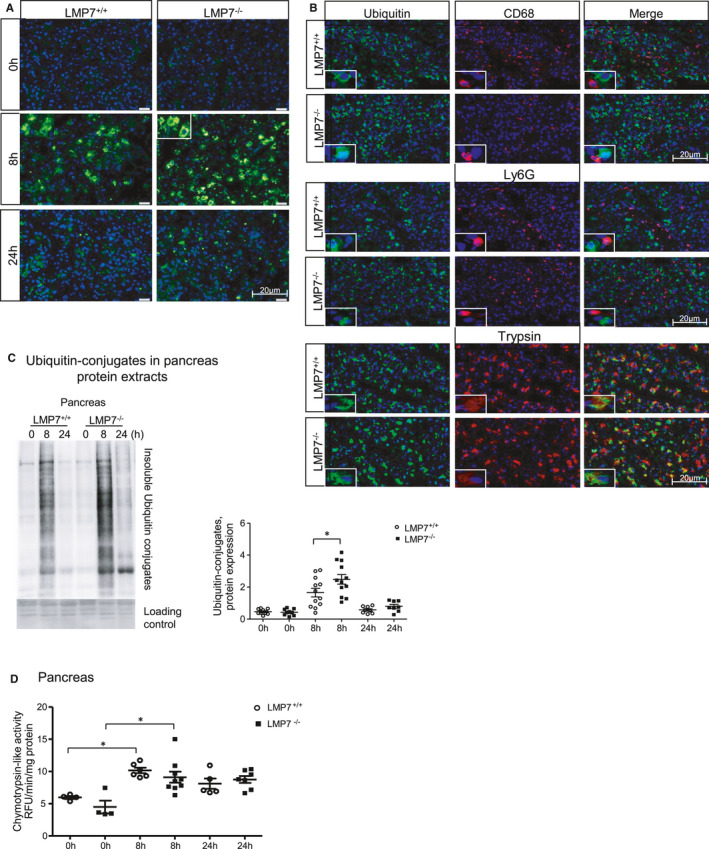
Impairment of ubiquitinated protein degradation in pancreatic acini from LMP7^−/−^ mice. Immunofluorescence in pancreas sections performed using ubiquitin A) alone or B) combined with CD68, Ly6G or trypsin antibodies. DAPI was used as nuclei staining. Scale bars, 20 µm. Image representative of five independent experiments. C, Higher accumulation of insoluble ubiquitin conjugates was quantified by Western blotting, and its ratio to a loading control (ponceau staining) was estimated by densitometry. (n = 8‐12). D, Proteasome‐like chymotrypsin activity was measured in pancreas homogenates using fluorescent substrate. Data are representative of independent experiments and expressed as means ± SEM. 0 h (n = 4), 8 h (n = 6‐9) and 24 h (n = 5‐7). *P < .05

In order to elucidate the molecular mechanisms associated with the exacerbated disease phenotype of β5i/LMP7^−/−^ mice during pancreatitis, we examined the immunoproteasome function by immunofluorescence and Western blotting; specifically, we analysed the profile of ubiquitin‐protein conjugates. First, we visualized the protein conjugates in the pancreas by immunofluorescence using an anti‐ubiquitin antibody. Our data showed an increased accumulation of ubiquitin‐modified proteins at 8 hours in the absence of β5i/LMP7. Next, we determined whether the ubiquitin‐protein conjugates were localized in pancreatic and/or immune cells by co‐immunofluorescence experiments on pancreatic tissue. Strikingly, the conjugates were detected exclusively in acini but not in macrophages or neutrophils (Figure 4A,B). The accumulation of ubiquitin‐modified proteins was about 1.5‐fold higher in the absence of the immunoproteasome, which was quantified by Western blotting in insoluble fractions of pancreas homogenates (Figure 4C). To investigate an inhibitory effect of caerulein in the proteasome activity, which would lead to the accumulation of protein aggregates, we measured the chymotrypsin‐like activity of the proteasome 20S core in pancreatic tissue homogenates. We demonstrated an increase in the proteasome chymotrypsin‐like activity with slightly decreased activity after caerulein‐induced pancreatitis in the LMP7‐deficient mice (Figure 4D). These findings suggest that an impairment of immunoproteasome function has an impact on protein degradation during pancreatitis, which it is not due to proteasome .

In addition to the ubiquitin‐dependent protein degradation pathway, we assessed whether the absence of β5i/LMP7 also had an influence on autophagy. Autophagy dysregulation has been identified as one key mechanism involved in the pathogenesis of pancreatitis. LC3‐II is the lipidated form of its cytosolic counterpart (LC3‐I) and is required for autophagosome formation. Sequentially, it engulfs cellular material for degradation and fuses with lysosomes, comprising the entity, in which protein cleavage occurs.^36^ Analysis of LC3‐II protein levels showed similar activation in the absence of β5i/LMP7, inferring that the induction of autophagy was not changed in our model (Supplementary Figure 3) and does not depend on the immunoproteasome.

### 3.5 Sustained modulation of the unfolded protein response in β5i/LMP7^−/−^ mice

**FIGURE 5:**
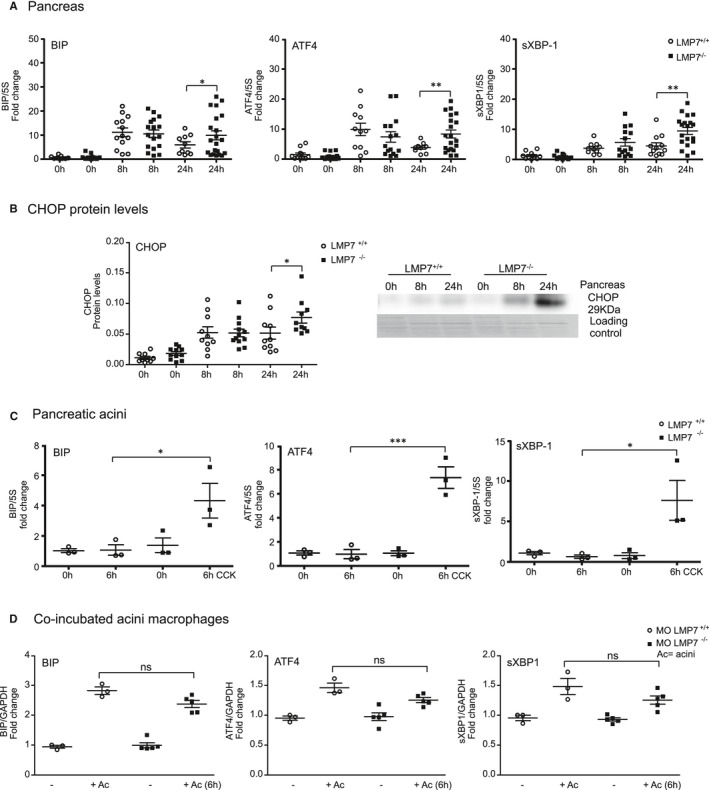
Stronger induction of ER stress transcripts in acute pancreatitis and isolated acini from LMP7^−/−^ mice. ER stress transcripts levels of BIP, ATF4 and sXBP‐1 were determined by real‐time qPCR using specific primers and 5S or GAPDH as housekeeping. A, RNA total from pancreas after pancreatitis; LMP7^+/+^ (n = 10‐13) and LMP7^−/−^ groups (n = 10‐21). B, CHOP protein expression was assessed by Western blotting in the pancreas (n = 9‐12). C, Isolated pancreatic acini stimulated with 1 µM of CCK for 6 h (n = 3). D, Co‐culture of acini and macrophages cells for 6 h at 37℃ (n = 3‐5). Data are illustrative of independent experiments and expressed as means ± SEM. *P < .05; **P < .01; ***P < .001

The expected consequences of immunoproteasome impairment would be delayed protein degradation, leading to protein accumulation within the ER.^37^ Thus, we hypothesized that immunoproteasome deficiency may result in increased ER stress and consequently, turn‐on of the UPR signalling pathways. To address the UPR response, we performed quantitative real‐time PCR in the pancreas to determine the expression of BIP and the transcription factors ATF4 and sXBP1, which are sensor and downstream targets of the UPR, respectively (Figure 5A). LPM7 deficiency had no effect on the transcript regulation in the acute phase (8 hours) of the disease. Nevertheless, there was a prolonged induction of the transcripts at 24 hours in the absence of β5i/LMP7, with an up to twofold increase over littermate controls. In addition, protein levels of C/EBP homologous protein (CHOP) (Figure 5B), which is a downstream target of the ATF4 and sXBP1 pathways, were significantly enhanced at 24 hours in the pancreas of β5i/LMP7^−/−^ mice. Subsequently, the ER stress response was also investigated in in vitro experiments using CCK‐treated pancreatic acini and co‐culture of acini and BMDM (Figure 5C,D). There was a clear induction of ER stress response transcripts BIP, ATF‐4 and sXBP‐1 in both cell types, but only acini underwent an altered regulation in the absence of β5i/LMP7. In addition, we verified whether the UPR dysregulation in LMP7‐deficient mice would also happen with another ER stress inducer, for example thapsigargin. Likewise to CCK treatment, we observed a significant twofold higher up‐regulation of ER stress transcripts, such as BIP, ATF4 and CHOP, in the absence of the immunoproteasome (Supplementary Figure 4). These observations confirm the importance of a functional immunoproteasome in regulating UPR induction.

### 3.6 Decreased oxidative phosphorylation is associated with acinar cell death in β5i/LMP7^−/−^ mice

**FIGURE 6:**
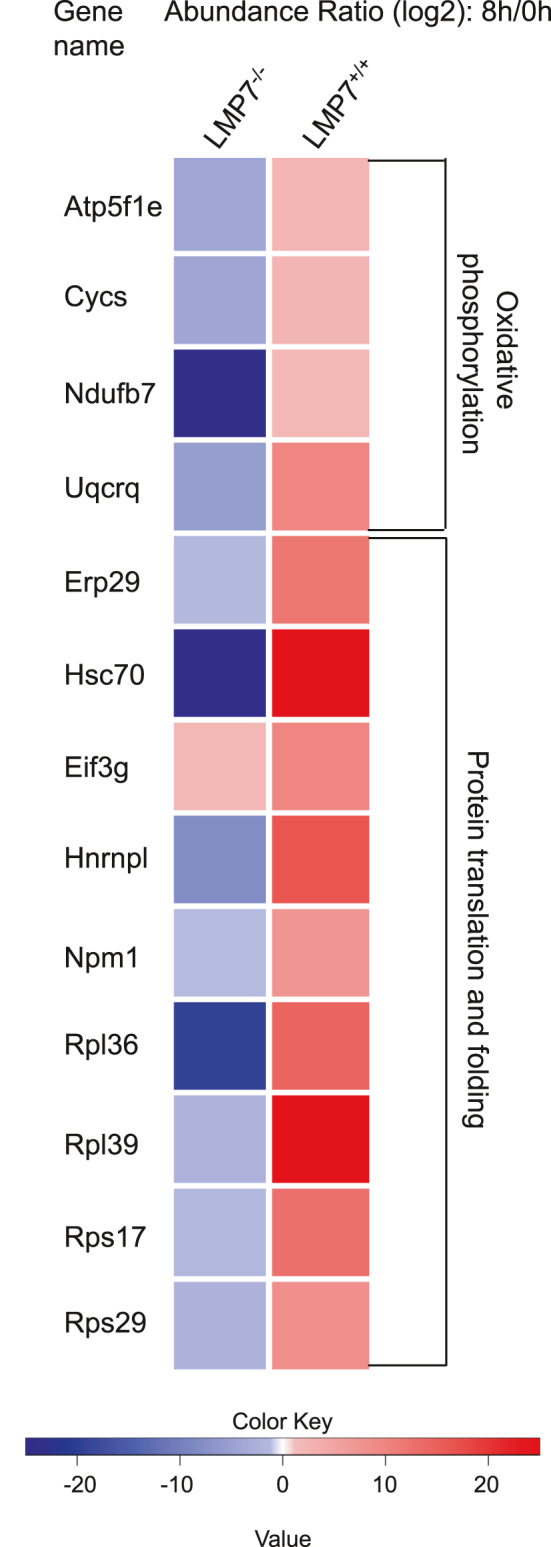
Differential protein content from pancreatic insoluble fractions of littermate controls and LMP7^−/−^ mice. A total of 7 μg of protein resuspended in 8 M urea/ 2 M thiourea was prepared and digested with trypsin using a bead‐based SP3 protocol. Measurements were performed on a QExactive HF mass spectrometer. Abundance ratios were calculated as the median of all possible pairwise peptide ratios calculated between replicates of all connected peptides. The heat map shows the significant abundance ratio from 8 h vs 0 h samples. Data represent independent experiments and are shown as means ± SEM. *P < .05. n = 3 (0 h), n = 4 (8 h)

To characterize the protein content during acute pancreatitis, we performed mass spectrometry analysis using pancreatic insoluble fractions from littermate controls and β5i/LMP7^−/−^ mice. Abundance ratio of samples from 8 hours vs 0 hour groups was calculated for each genotype and was plotted for selected proteins as a heat map (Figure 6). We confirmed imbalanced protein homeostasis by decreased regulation of protein translation and folding in immunoproteasome‐deficient mice. Moreover, we observed decreased abundance ratios of proteins from the oxidative phosphorylation metabolic pathway, suggesting ATP depletion and increased oxidative stress. A list of the proteins from the heat map can be found in Supplementary Table 1.

Overall, our data imply that the immunoproteasome plays a protective role in acinar cell homeostasis, since elevated cell death was observed in the absence of β5i/LMP7 in pancreatitis. The effect on pancreatic injury did not relate to changes in acinar cell regeneration, considering that the proliferation rate visualized by immunohistochemistry was not altered in the absence of β5i/LMP7 (Supplementary Figure 5). Therefore, the immunoproteasome appears to contribute to the clearance of ubiquitin‐protein aggregates, resolution of inflammation and the reversal of UPR activation in pancreatitis.

## 4 DISCUSSION

In this study, we report a protective role of the immunoproteasome in pancreatitis and a beneficial effect on pancreatic injury resolution. The expression of the immunoproteasome subunits is known to be regulated not only in immune cells but also in non‐immune cells, for example in pancreatic beta cells, muscle cells and adipocytes.^38^, ^39^, ^40^ Previous reports have demonstrated the importance of this proteasome isoform in cell differentiation and protein homeostasis.^37^, ^41^ Our work provides evidence for a role of the immunoproteasome in pancreatic acinar cell homeostasis and tissue damage. Notably, the β5i/LMP7 subunit but not the β5 constitutive subunit was up‐regulated in an in vitro and an in vivo model of pancreatitis. Increased β5 protein levels in acini from β5i/LMP7‐deficient mice were observed as a compensatory mechanism, as seen before in other cell types.^42^, ^43^ However, from the data obtained in this study, the immunoproteasome represents a major proteasome type involved in a rapid and efficient response to inflammation and ER stress during pancreatitis.

In the development of pancreatitis, well known as a primarily sterile disease, two major players have been described in the past. Injured acinar cells release cellular components, for instance free ATP, histones or DNA, which act as damage‐associated molecular patterns (DAMPs). These elicit the recruitment of macrophages and neutrophils into the pancreas, which then trigger inflammation and are associated with tissue injury. DAMPs are recognized by Toll‐like receptors within phagocytic macrophages, which activate the inflammasome complex and the NF‐κB signalling pathway, resulting in release of pro‐inflammatory cytokines IL‐1β, TNF‐α and IL‐6.^12^, ^34^ Although acinar cells per se can undergo NF‐kB transcription factor activation and secrete pro‐inflammatory cytokines and chemokines, such as IL‐6, TNF‐α and MCP‐1,^44^ macrophages are the main source of cytokines production.^34^ The up‐regulation of the β5i/LMP7 subunit in acini and in macrophages might be linked to Toll‐like receptor pathways, as shown by other groups during infection and ethanol treatment.^45^, ^46^ In inflammatory diseases, several reports have associated β5i/LMP7 inhibition or deficiency with diminished cytokine production.^47^, ^48^, ^49^ Here, we show an opposite effect in which immunoproteasome deficiency correlates with an increased production of pro‐inflammatory cytokines where acini and macrophages are affected. Over the last decade, various studies have found an association between oxidative stress and acinar cell damage, with neutrophils as the central player in this process.^50^ The higher extent of neutrophil infiltration in the absence of β5i/LMP7 subunit, accompanied by increased myeloperoxidase activity, may account for stronger oxidative stress. In line with this, Seifert et al have previously shown a primordial role of the β5i/LMP7 subunit in preserving cell viability upon cytokine‐induced oxidative stress.^37^ Our work suggests a functional link between immunoproteasome, ER stress and inflammation, since pancreatic acinar cells from β5i/LMP7‐deficient mice have a stronger ER stress response, evoking pronounced pro‐inflammation.

It is well established that acini, in response to supraphysiological secretagogue stimulation, develop ER stress and subsequently activate UPR.^51^ In turn, UPR up‐regulation has been recognized as a protective mechanism against acinar cell injury in pancreatitis.^14^ The UPR restores ER protein homeostasis through inhibition of protein synthesis, enhancement of protein folding and degradation mechanisms, such as autophagy and the UPS.^17^ Recently, the UPR and the immunoproteasome have appeared as pivotal networks in the maintenance of protein homeostasis and cell fate control.^52^, ^53^ In our work, we show that the immunoproteasome is crucial for limiting UPR activation in isolated pancreatic acini. The UPR encircles stress response signalling pathways triggered by accumulation of misfolded or damaged proteins in the ER lumen.^54^ Although some studies did not report differences in the capacities of the constitutive‐ and the immunoproteasome to degrade ubiquitinated protein targets,^43^, ^55^ our work supports an essential role of the immunoproteasome in adjusting this process and synchronizing UPR activation in pancreatitis.

Interestingly, type I interferonopathies, characterized by excessive type I interferon (IFNα/β) production, have been associated with proteasome impairment. Mutations in PSMB8 encoding β5i/LMP7 cause a rare autoinflammatory disease (proteasome associated autoinflammatory syndromes, PRAAS), which is accompanied by transcription of IFN‐stimulated genes, such as CXCL‐10. In this scenario, β5i/LMP7 deficiency leads to an imbalance of the intracellular protein homeostasis, eliciting accumulation of ubiquitinated proteins and UPR activation, which is accompanied by a type I interferon signature.^56^, ^57^ Unbalanced UPR due to proteasome dysfunction has been implicated in the innate immune response, with the immunoproteasome playing a central role in the regulation of inflammation and UPR. The crosstalk between UPR and type I IFN signature has been proposed to evoke a sterile inflammation in the event of increased ER stress levels.^57^, ^58^, ^59^, ^60^ Our data align with these findings and provide further insight in the role of the immunoproteasome in acinar cell protein homeostasis and into the connection of inflammation and UPR in acute pancreatitis.

As a consequence of UPR dysregulation, represented by a prolonged time span for activation of sensor proteins and downstream targets, cell death appears to be the outcome.^53^ Immunoproteasome dysfunction correlates with sustained pancreatic damage, which is accompanied by increased acinar cell death, namely apoptosis and necrosis. These observations support previous findings, implying that the UPR is to a certain extent protective via restoring protein homeostasis. Nevertheless, its sustained activation may lead to cell death.^18^, ^61^ One of the proposed mechanisms involves the ATF4‐CHOP signalling pathway, a downstream target of the PERK stress sensor. In acute pancreatitis, C/EBP homologous protein (CHOP) is induced by ER stress and was linked to apoptosis by inflammasome activation and IL‐1β secretion.^62^ In addition, CHOP was also shown to regulate IL‐6 transcription via STAT signalling, which leads to a sustained inflammation in case of protease impairment.^58^ Consistent with these findings, the absence of the immunoproteasome links a stronger CHOP activation with higher apoptosis rates in the course of pancreatitis. The proteomic data show that impaired protein homeostasis and increased cell death are also correlated with diminished oxidative phosphorylation and protein translation/folding. Thus, we identified a role of the immunoproteasome by responding efficiently to ER stress, reducing proteotoxic stress levels and restoring acinar cell viability.

Selective LMP7 inhibition has been suggested as a therapeutic modality in autoimmune diseases, such as rheumatoid arthritis, since it induces an anti‐inflammatory response at lower doses than nonselective inhibitors, such as bortezomib, and reduces serum autoantibody levels, blocking disease progression.^47^ In type I diabetes, abnormal self‐antigen production leads to autoimmunity in patients and animal models. Due to its role in the generation of MHC‐presented autoantigenic epitopes, the immunoproteasome might play a part in the autoimmune destruction of insulin‐producing pancreatic β‐cells.^38^ In acute pancreatitis, the balance of UPR and the maintenance of acinar cell protein homeostasis are important cellular processes involved in the severity of tissue injury. The immunoproteasome appears to be a critical proteolytic complex in mitigating the pro‐inflammatory response and preventing exacerbated pancreatic injury acute from pancreatitis. Future studies will be needed to identify pharmaceutically usable agents that improve or maintain the function of the immunoproteasome in order to translate this novel treatment target into a therapeutic modality.

## CONFLICT OF INTEREST

The authors have declared that no conflict of interest exists.

## AUTHOR CONTRIBUTIONS

**Laura Leticia de Freitas Chama:** Conceptualization (lead); Formal analysis (lead); Investigation (lead); Methodology (lead); Project administration (lead); Validation (lead); Visualization (lead); Writing‐original draft (lead). **Frederic Ebstein:** Conceptualization (supporting); Methodology (supporting); Resources (supporting); Writing‐review & editing (supporting). **Birthe Wiesrecker:** Investigation (supporting); Methodology (supporting); Writing‐review & editing (supporting). **Preshit Wagh:** Methodology (supporting); Writing‐review & editing (supporting). **Elke Hammer:** Formal analysis (supporting); Investigation (supporting); Methodology (supporting); Writing‐review & editing (supporting). **Frank Ulrich Weiss:** Funding acquisition (supporting); Resources (supporting); Writing‐review & editing (supporting). **Heike Junker:** Methodology (supporting); Resources (supporting); Writing‐review & editing (supporting). **Maja Studencka‐Turski:** Resources (supporting); Writing‐review & editing (supporting). **Markus Lerch:** Conceptualization (lead); Funding acquisition (lead); Writing‐review & editing (supporting). **Elke Krueger:** Conceptualization (lead); Funding acquisition (supporting); Methodology (supporting); Resources (supporting); Writing‐review & editing (supporting). **Matthias Sendler:** Conceptualization (supporting); Funding acquisition (supporting); Methodology (supporting); Writing‐review & editing (supporting).

## Supporting information

Fig S1‐5

Table S1

Supplementary Material

## Data Availability

The data that support the findings of this study are available from the corresponding author upon reasonable request.
