# Supplementary material for: Immunoproteasome impairment via β5i/LMP7‐deletion leads to sustained pancreatic injury from experimental pancreatitis
Source: J Cell Mol Med. 2021 Jun 15;25(14):6786–99. doi: 10.1111/jcmm.16682 (PMC8278072; doi:10.1111/jcmm.16682)
Supplement: Supplementary file 1 — Fig S1‐5 [file JCMM-25-6786-s003.pdf]

Suppl. Figure 1: Intracellular protease activation and cell death rate in living acinar cells.

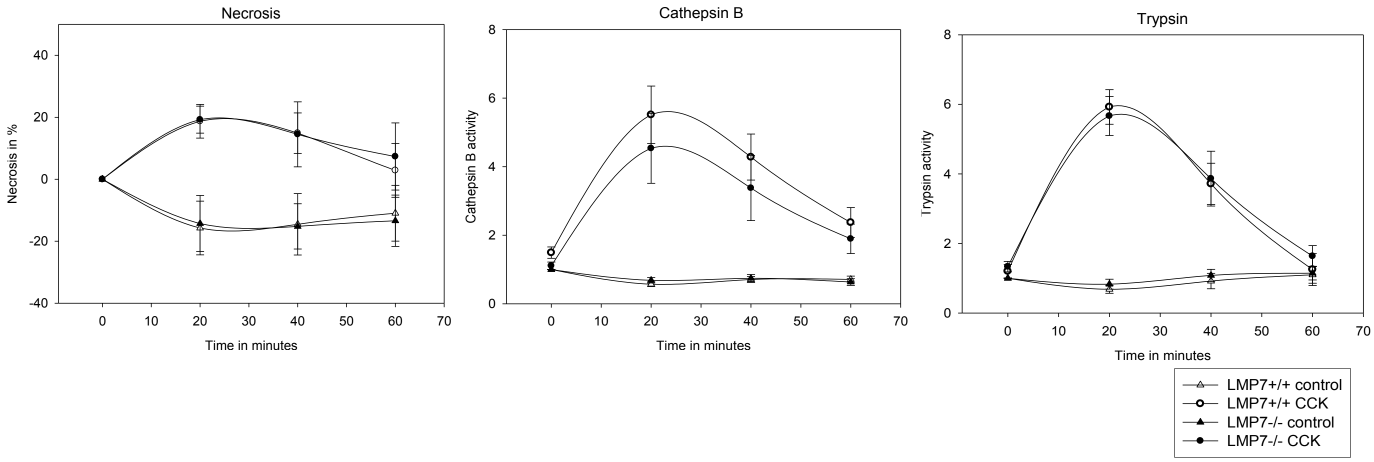

Figure S1: Intracellular protease activation and cell death rate in isolated acini. Trypsin and CTSB activation as well as cellular necrosis were not primarily affected by LMP7 depletion. Acini were supramaximal stimulated with CCK and both enzymatic activities assessed with fluorescent substrates in a time-course reaction. Cell death was measured by propidium iodide inclusion. Data are representative of independent experiments and visualized by means ± SEM. (n=8-10).

Suppl. Figure 2: Expression and production of pro-inflammatory cytokines *in vitro*

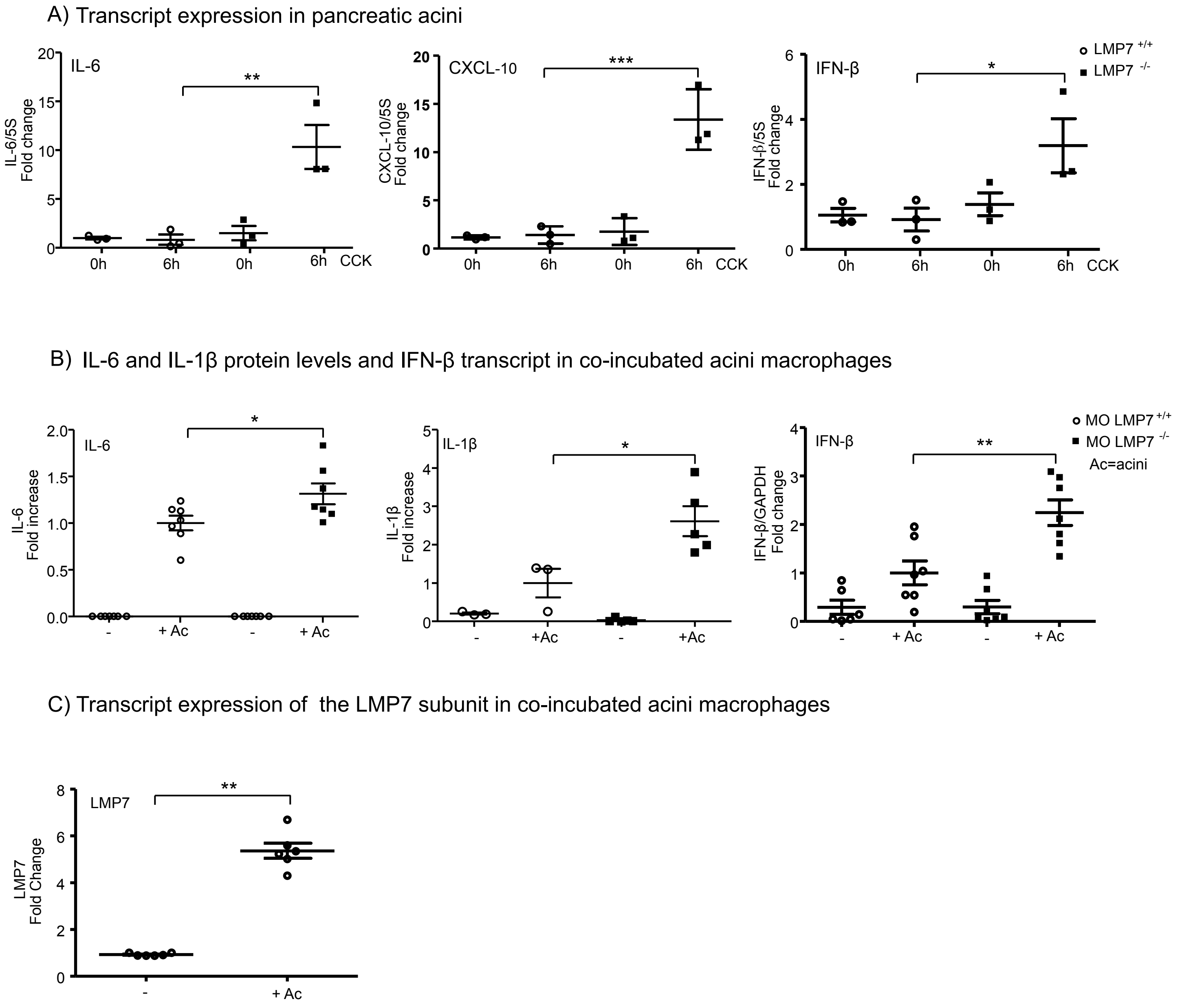

Figure S2: **Production of pro-inflammatory cytokines *in vitro*.** The corresponding transcripts were assessed by real-time qPCR. 5S and GAPDH were used as housekeeping for acini and macrophages, respectively. A) Isolated acini were incubated with 1μM of CCK for 6h at 37°C. B) Co-exposed acini macrophages were incubated in cell culture for 9h at 37°C. Secreted levels of IL-6 and IL-1β cytokines were measured in the supernatant by CBA and ELISA, respectively, and IFN-β transcript expression by real-time qPCR. Data were normalized respective to the LMP7$^{+/+}$ 8h group. Data are representative of independent experiments and expressed as means ± SEM (n=3-7). *p<0.05, **p<0.01, ***p<0.001.

Suppl. Figure 3: Absence of LMP7 subunit has no influence on autophagy activation

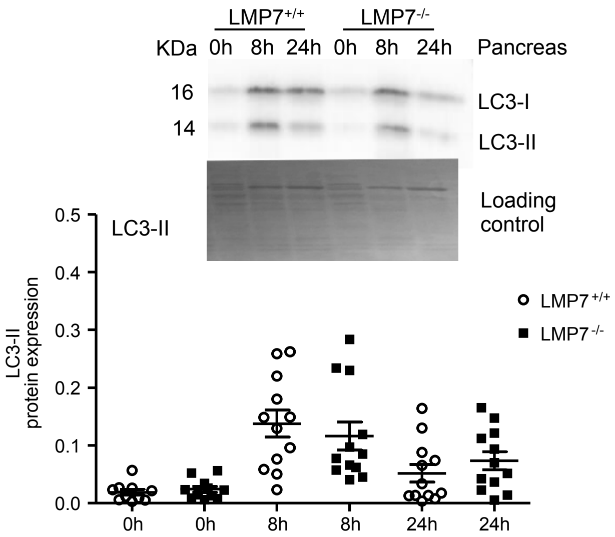

Figure S3: Absence of LMP7 has no influence on autophagy activation. LC3 isoforms were detected in pancreas homogenates by western blotting analyses. LC3-II ratio to a loading control (ponceau staining) was calculated by densitometry. No differences were observed in the experimental conditions. Data are representative of independent experiments and expressed as means ± SEM. (n=10-12).

Suppl. Figure 4: The thapsigargin-induced ER stress response is stronger in acinar cells of LMP7-deficient mice

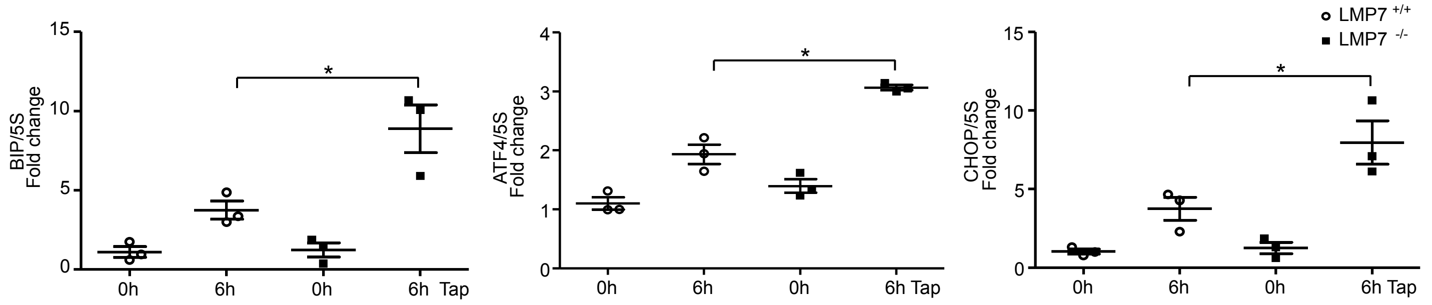

Figure S4: Thapsigargin-induced ER stress response is stronger in pancreatic acini of LMP7$^{-/-}$ mice. Isolated acini from littermate controls and LMP7-deficient mice were treated with 1µM thapsigargin for 6h. ER stress transcripts levels of BIP, ATF4 and CHOP were determined by real-time qPCR using specific primers and 5S as an internal control. Data are illustrative of independent experiments and expressed as means ± SEM (n=3). *$p<0.05$.

Suppl. Figure 5: Acinar cell proliferation is unaltered in LMP7$^{-/-}$ mice

Immunohistochemistry in the pancreas with anti-Ki67 proliferation marker

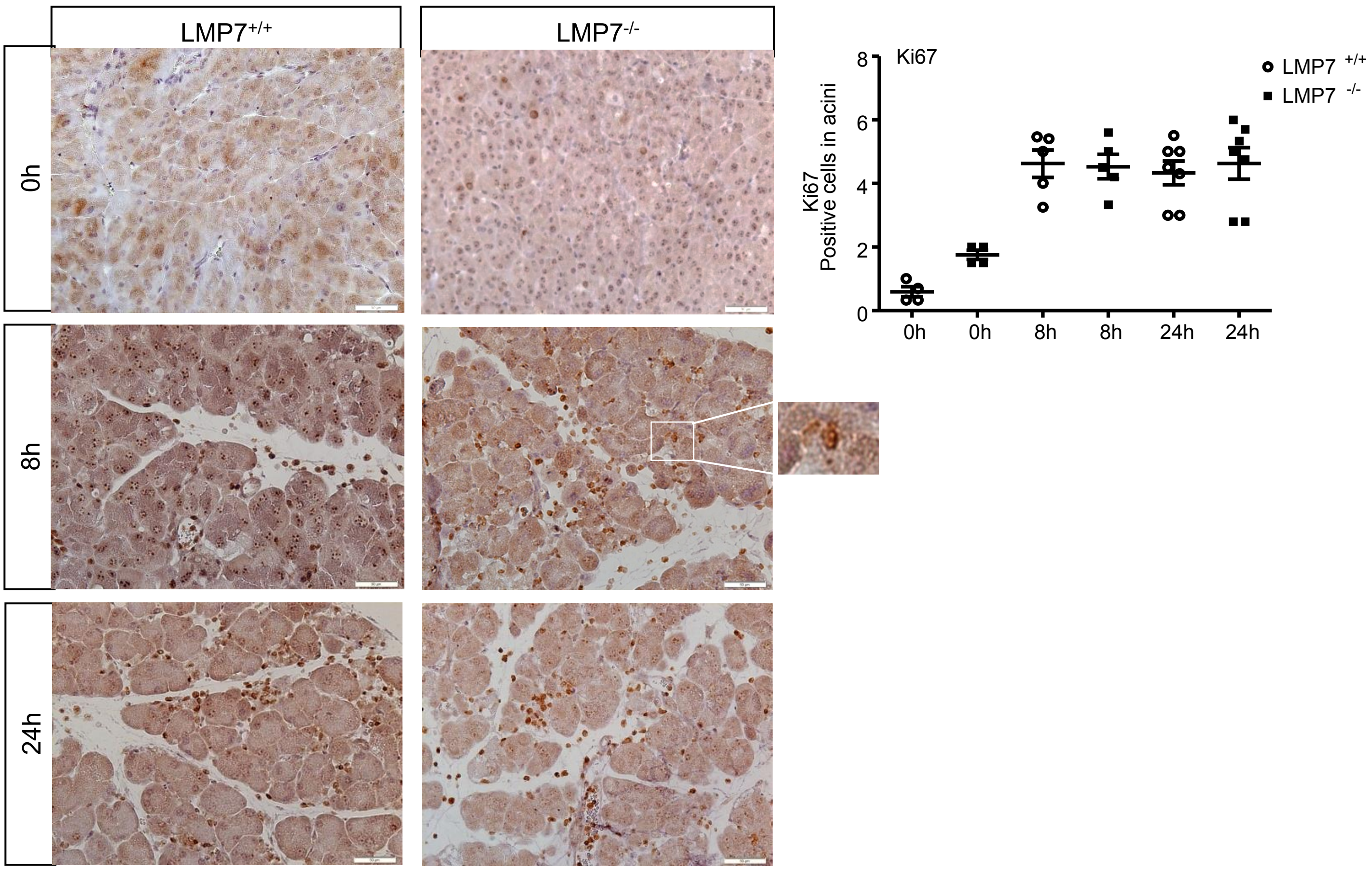

Figure S5: Acinar cell proliferation is unaltered in LMP7$^{-/-}$ mice. A) Ki67 proliferation marker was assessed in the pancreas by immunohistochemistry using DAB as substrate (brown-black stain). Hematoxylin accounted for nuclei staining and the positive staining from inflammatory cells was excluded from the quantification. Scale bars, 50µm. Image representative of five independent experiments. Data are expressed as means ± SEM. (n=5-7).
