# Supplementary material for: Immunoproteasome impairment via β5i/LMP7‐deletion leads to sustained pancreatic injury from experimental pancreatitis
Source: J Cell Mol Med. 2021 Jun 15;25(14):6786–99. doi: 10.1111/jcmm.16682 (PMC8278072; doi:10.1111/jcmm.16682)
Supplement: Supplementary file 3 — Supplementary Material [file JCMM-25-6786-s001.doc]

**Supplementary Methods**

**Biochemical assays**

Trypsin substrate (R110-(CBZ-Ile-Pro-Arg)2 was purchased from Life Technologies (Carlsbad, CA, USA); Chymotrypsin (Suc-AAPF-AMC) substrates was obtained from Bachem AG (Bubendorf, Switzerland).

**Proteasome Chymotrypsin-like Activity Assay**

Pancreas were homogenized in native-lysis buffer (10 mM Tris pH 7,5, 25 mM KCl, 10 mM NaCl, 1 mM MgCl^2^, 10% Glycerol, 2 mM ATP, 2 mM DTT) and tissue lysis was performed by 4 rapid freeze/thaw cycles using liquid nitrogen. Next, we centrifuged the samples for 15 min at 13.000 rpm, at 4°C. Protein concentration from supernatant was determined using Pierce BCA assay and bovine serum albumin as a standard protein. The proteasome chymotrypsin-like activity from the 20S core was determined using the fluorescent substrate 0.2mM Suc-LLVY-AMC (I-1395 Bachem) over 1h of reaction. Fluorescence signal was detected using a plate reader with excitation and emission wavelengths of 360nm and 460nm. The enzymatic activity was normalized by protein concentration.

**Imaging analyses**

For the immunofluorescence experiments, the following primary antibodies were used: anti-FK2 (cat: BML-PW0150; Enzo life Sciences, Farmingdale, NY), anti-ubiquitin (cat: 43124, Cell Signaling Technology, Danvers, MA, USA), anti-CD68 (cat: ABIN181836, antibody online; Aachen, Germany), anti-Mrc1/CD206 (cat: OASA05048; Aviva Systems Biology, San Diego, CA), anti-Ly6g (cat: 25377, Abcam, Cambridge, MA). The secondary IgG antibodies (fluorophores Cy3 and Alexa488) were purchased from Jackson Immunoresearch (Pennsylvania, USA). The primary antibody dilution variated of 1:200 up to 1:400 with overnight incubation at 4ºC and 1:400 for the secondary staining with 2h incubation at room temperature. DAPI (4′,6-diamidino-2-phenylindole; 1:1000) was used as nuclei staining and fluorescent mounting medium (DAKO) to cover the slides before imaging acquisition.

**Transcript expression by quantitative Real‐time PCR**

RNA extraction was performed following the manufacturer’s instructions (Thermo Fisher Scientific), using RNAse‐free labware. The RNA concentration was measured spectrophotometrically at 260 nm. Next, oligo‐dT and random hexamer primer (RH; cat: SO181) were used to generate cDNA from 1-2µg RNA using M-MLV reverse transcriptase (cat: 28025013). Reactions were performed in an Applied Biosystems QuantStudio 7 Flex Real Time System using SYBR Green PCR Master Mix (cat: 4334973), according to the manufacturer's recommendations.

**Measurement of protein levels by western blotting**

Specifically, insoluble ubiquitin-protein conjugates were detected after running 8% gels and PVDF membrane whereas 12% gels and nitrocellulose membrane were used for β5i/LMP7 and CHOP and 15% for LC3-II analyses. Blocking was done with 1% Roti-Block (cat: A151.1; Carl Roth, Karlsruhe, Germany) before primary antibody overnight incubation at 4ºC at the following concentrations: anti-LMP7 (1:100000), anti-β5 (1:2000; cat: 3330, Abcam), anti-ubiquitin (1:5000; FK2, BML-PW0150, Enzo Life Sciences), anti-CHOP (1:2000; 2895, Cell Signaling) and anti-LC3 (1:2000; 2775, Cell Signaling). All membranes were incubated with secondary horseradish peroxidase conjugated antibody (1:10000; GE Healthcare, Chicago, Illinois, USA) for 1-2 h at room temperature. Afterwards, the membranes were developed by chemiluminescence with SuperSignal™ West Femto-ECL-substrate (cat: 34095; Thermo Fisher Scientific) and imaging capture was acquired by Fusion equipment.

**LC‐MS/MS measurements and data analysis**

A total of seven μg of protein resuspended in 8 M urea/ 2 M thiourea was reduced (2.5 mM DTT ultrapure, Invitrogen, for 15 min at 37 °C) and alkylated (10 mM iodacetamide for 30 min at 37 °C, Sigma Aldrich). Subsequently protein was precipitated and digested with trypsin in an enzyme to protein ratio of 1:25 on SP3 beads.

A QExactive HF mass spectrometer (Thermo Scientific, Bremen, Germany) with online coupling to an Ultimate 3000 nano HPLC (Dionex/ Thermo Scientific, Waltham, MA, USA) was used for LC‐MS/MS analysis. Chromatographic separation of tryptic peptides was achieved by 120 min-linear gradients with increasing acetonitrile concentration from 5-25 % in 0.1% acetic acid on a reverse phase column (Accucore 150-C18, 25 cm x 75 μm, 2,6 μm C18, 150 Å) at a constant temperature of 40 °C and a flow rate of 300 nL/min. The MS scans were carried out in a *m/z* range of 333 to 1650 *m*/*z*. Data was acquired with a resolution of 60,000 and an AGC target of 3 x 10E6 at maximal injection times of 20 ms. The top 15 most abundant isotope patterns with charge ≥2 from the survey scan were selected for fragmentation by high energy collisional dissociation (HCD) with a maximal injection time of 25 ms, an isolation window of 1.4 *m/z*, and a normalized collision energy of 27.5 eV. Dynamic exclusion was set to 20 s. The MS/MS scans had a resolution of 15,000 and an AGC target of 1 x 10E5.

Protein identification and quantification was performed in Proteome Discoverer software (version 2.4) (Thermo Scientific). For database search the Sequest algorithm and the Uniprot/Swissprot database limited to murine entries (version 01_2020) were used and the following search parameters applied: 10 ppm of mass tolerance for precursor ions and 0.02 Da for fragment ions; specified enzyme: trypsin; missed cleavages: 2; fixed modification: carbamidomethylation at cysteine; variable modification: methionine oxidation; ubiquitinylation (GG) at lysine; loss of methionine or acetylation, or both modifications at the protein N-terminus. Only high‐confidence peptides (FDR) <1%) based on the target‐decoy approach were considered for protein group definition. Differential abundance was calculated as the median of all possible pairwise peptide ratios calculated between replicates of all connected peptides. Background based T-test was used to calculate p-values and adjusted p-values. Proteins with an adjusted p-value < 0.05 were considered as differentially abundant.
